# Case Report: CMV Infection and Same Mechanism-Originated Intestinal Inflammation Compatible With Bowel/Crohn's Disease Is Suggested in ATP4A Mutated-Driven Gastric Neuroendocrine Tumors

**DOI:** 10.3389/fmed.2021.553110

**Published:** 2021-04-06

**Authors:** Oriol Calvete, José Reyes, Javier Benítez

**Affiliations:** ^1^Human Genetics Group, Spanish National Cancer Research Center (CNIO), Madrid, Spain; ^2^Network of Research on Rare Diseases (CIBERER), Madrid, Spain; ^3^Grupo Español de Tumores Neuroendocrinos y Endocrinos, Madrid, Spain; ^4^Department of Gastroenterology, Hospital Comarcal de Inca, Balearic Islands Health Investigation Institute (IDISBA), Majorca, Spain

**Keywords:** gastric neuroendocrine tumor, ATP4A, co-occurring autoimmune disorders, cytomegalovirus, inflammatory bowel disease, Crohn's disease

## Abstract

Mutations in the ATP4A proton pump prevent gastric acidification and explain the chronic autoimmune gastritis scenario that conducts the gastric neuroendocrine tumor (gNET) formation. Here, we wanted to investigate the co-occurrence cytomegalovirus (CMV) infection and intestinal inflammation that presented all members of a family affected with gNET and carrying an *ATP4A* mutation. Intestinal inflammation persisted after CMV eradication and anemia treatment. The inflammation was compatible with a ileitis/Crohn's disease and was originated by the same autoimmune mechanism described in the tumorigenesis of gNETS. The same secondary disease but no the CMV infection was observed in all members affected with gNET and carrying the ATP4A mutation. Our results suggest that the *ATP4A* malfunction not only explained gNETs but also the co-occurring disease and opportunistic infections, which allowed to link autoimmune pathologies and gNETs in a unique mechanism. Our results open a new window to better understand not only gastric neoplasms formation but the co-occurring autoimmune disorders and the inflammatory mechanism that compose a premalignant scenario for other tumor formation. Our findings are important since contribute to describe the genetic landscape of the Inflammatory Bowel/Crohn's disease and alert clinicians to monitor patients with gastric neoplasms mediated by achlorhydria mechanisms for concomitant secondary pathologies.

## Introduction

Type I gastric neuroendocrine tumors (gNETs) arise from enterochromaffine-like (ECL) cells in patients with autoimmune chronic atrophic gastritis and give rise to hypergastrinemia and parietal cell (PC) atrophy that leads to gastric hyperplasia and achlorhydria, respectively. However, we described a homozygous mutation in the *ATP4A* gene (p.R703C) that prevented gastric acidification and was the main effector of progression of the gNETs. This contrasts with the classical model, where hypergastrinemia leads to achlorhydria (1). A knock-in (KI) mouse model for this *ATP4A* mutation was constructed in order to perform functional studies (2). This model confirmed the relation of the candidate gene with achlorhydria and gNET development, and served us to better understand the relation between impaired capability to export protons across the plasma membrane of PCs and tumor progression. We observed that the ATP4A^p.R703C^ mutation drove gastric achlorhydria, but also would impair the acid–base balance within PCs, affecting mitochondrial biogenesis and activating ROS signaling, which triggers caspase-3-mediated apoptosis of parietal cells (3). Recently, we studied a second family affected with gNETs and other autoimmune pathologies (hypothyroidism and rheumatoid arthritis). A cumulative effect of two mutations in a digenic model (*ATP4A* and *PTH1R* genes) explained the genetic landscape underlying the gNETs in this family. In addition, the *PTH1R* gene is involved in the regulation of Ca2+ metabolism and this explained the associated hypothyroidism and rheumatoid arthritis (4). Here, we present the monitoring results of the first family with the atypical and aggressive gNET caused by the ATP4A^p.R703C^ mutation (1) and the recent re-evaluation of the altered clinical parameters not involved in the gastric pathology.

## Materials and Methods

### Patients

**Figure 1:**
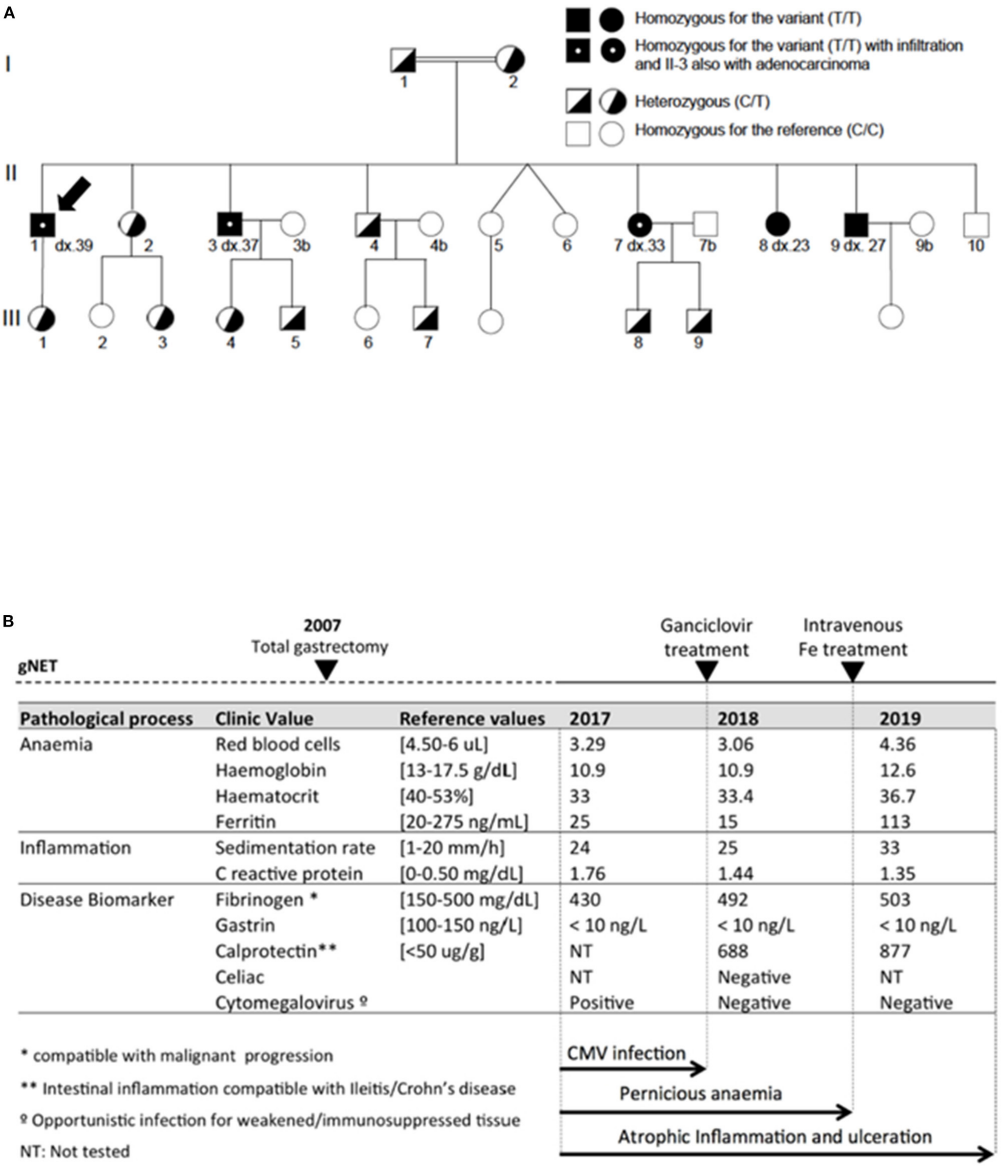
**(A)** Pedigree of studied family with complete segregation of the ATP4A^p.R703C^ mutation (C: Reference allele for the position; T: Alternative allele for the position). Black arrow shows the proband evaluated in this study. **(B)** Serologic information of proband patient regarding anemia, inflammation and disease markers measured in 2017, 2018, and 2019. CMV infection was eradicated after ganciclovir treatment. Anemia markers become partially restored after Fe treatment. Atrophic inflammation persisted in the 2019 evaluation.

A consanguineous family (the parents were cousins) from Majorca Island (Spain) with ten siblings was previously evaluated (1). Five of them were diagnosed (age of onset around 30 years in average) with type I gastric NETs (II-1, II-3, II-7, II-8, II-9). Three (II-1, II-3, and II-7) showed nodal infiltration and one (II-3) had a synchronous focus of gastric adenocarcinoma without nodal infiltration (T1b N0) (Figure 1A). All patients were treated with total gastrectomy, were negative in *MEN1* gene mutation studies by Sanger sequencing and for *H. pylori*. Both parents (I-1 and I-2) and siblings II-3, II-7 and II-9 were selected for WES, which uncovered the ATP4A mutation (in heterozygosis in parents and in homozygosis in affected siblings). Blood samples from the remaining healthy individuals in the family were also obtained for further study of the segregation of the variants (Figure 1A). Role of the ATP4A mutation was later validated in a KI mouse model (2). Informed consent was obtained for all patients. Serological and biochemical parameters were obtained from routine serum/blood tests.

### Immunohistochemistry Studies

Formalin-fixed paraffin-embedded (FFPE) tissue samples were obtained from monitoring biopsies of the proband. FFPE blocks were cut into 5-μm-thick sections and stained with Hematoxylin and Eosin (H&E) for light microscopy examination. FFPE blocks were cut into 5-l m-thick sections for immunohistochemistry (IHC) studies. Inflammation of the ileum was tested with anti-cytokeratin 7 antibody from DAKO (ref: M7018). Mitochondrial activity in the inflamed area was tested with anti-Pyruvate Carboxylase antibody from NOVUS Biologicals (Ref: NBP1-49536) following the manufacturers' instructions.

## Case Presentation

A consanguineous family with gNETs was previously studied. The proband of this study (Figure 1A) was diagnosed in 2007 (dx.39) with four well-differentiated foci of gNETs of 1.7, 1.0, 0.9, and 0.7 cm in size that were infiltrating the submucosal layer (pT1b). The tumors were immunoreactive for the general neuroendocrine markers but also had a component with glandular growth, which was morphologically classified as an intestinal type adenocarcinoma (G1/4) (5). Perineural and lymph node invasion (size: 0.4 cm) was also reported for this patient. Total gastrectomy was performed to prevent adenocarcinoma metastasis.

## Results and Discusion

**Figure 2:**
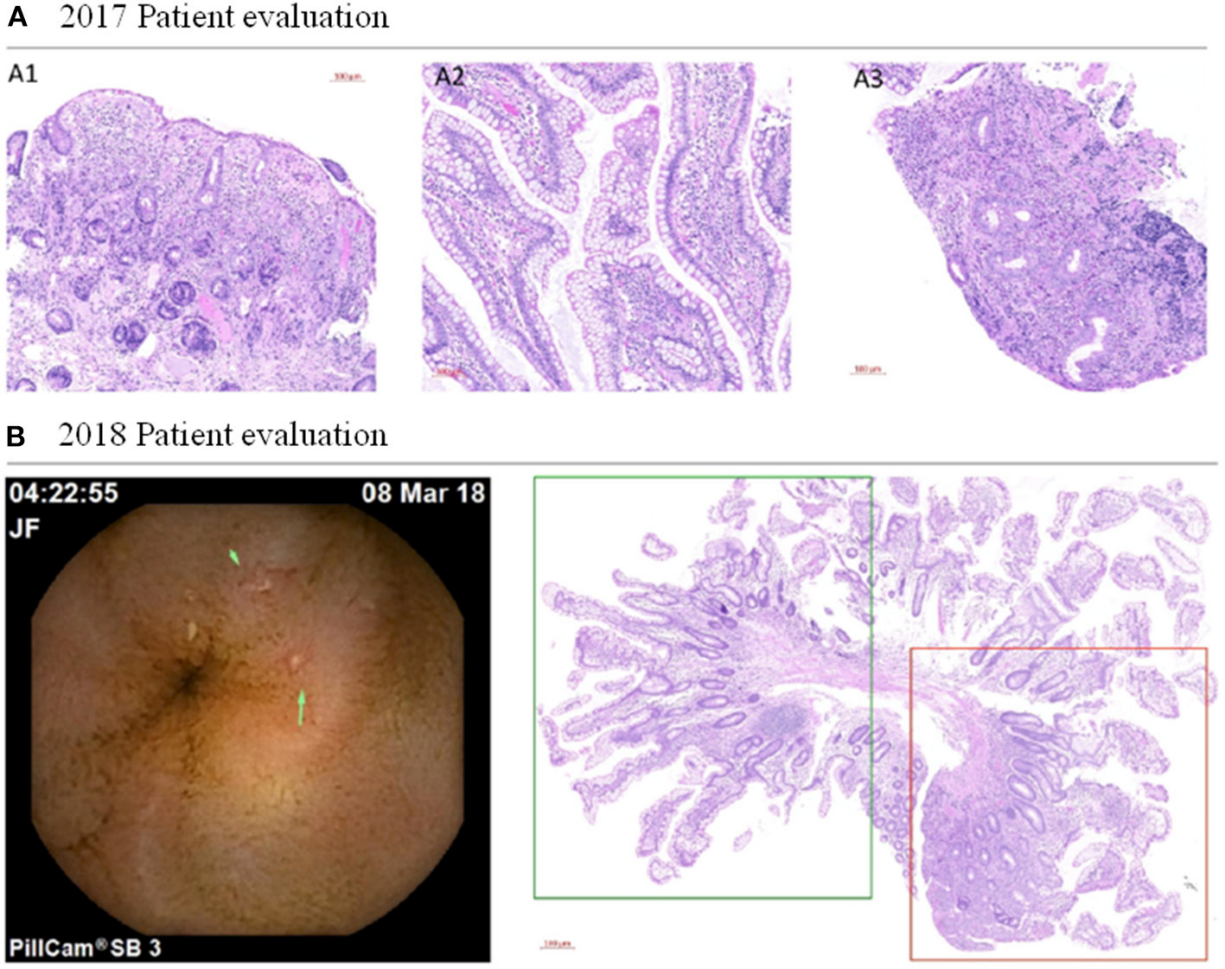
Clinicopathologic evaluation of the patient. **(A)** Histopathologic evaluation of the patient's 2017 biopsy. Intestine biopsy revealed atrophic and flattened microvilli with reduction of goblet cells (A1), microvilli oedema and cryptitis (A2), and inflammatory infiltrate (A3). Scale bar: 100 μm. **(B)** Histopathologic evaluation of the patient's 2018 biopsy. Left panel: The endoscopy uncovered aphthous ulcers (green arrows) in the small intestine even without cytomegalovirus infection. Right panel: haematoxylin and eosin staining of biopsied tissue shows moderate microvilli atrophy in the normal intestinal tissue of the patient (boxed in green) and inflammation process (boxed in red). Scale bar: 500 μm.

**Figure 3:**
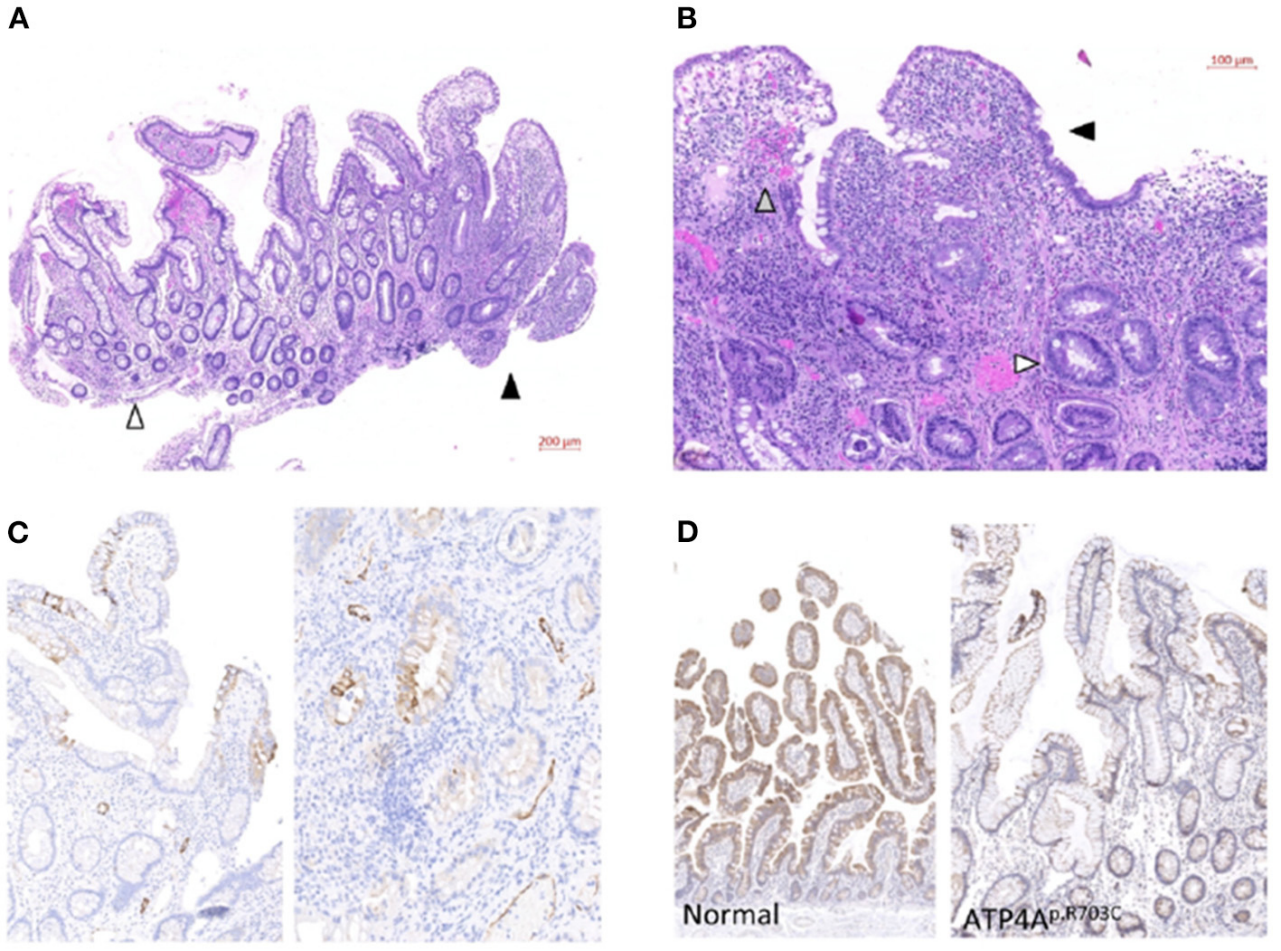
Clinicopathologic evaluation of the patient. **(A)** Representative H&E staining of 2019 ileum biopsy. White arrowhead: moderate microvilli atrophy in normal intestinal tissue of the patient. Black arrowhead: severe inflammation process in a biopsied aphthous ulcer. Scale bar: 200 μm. **(B)** Detailed evaluation of inflamed area shows atrophic and flattened microvilli with strong reduction of goblet cells (black arrowhead) and oedema and cryptitis (gray arrowhead). Reactive glands (white arrowhead) and inflammatory infiltrate with abundant neutrophils and eosinophils are observed in the whole mucosa. Scale bar: 100 μm. **(C)** Two different representative areas of the 2019 patient intestine biopsy stained with anti-cytokeratin7 antibody. Scale bar: 100 μm. **(D)** Representative immunohistochemical staining with anti-Pyruvate Carboxylase antibody of a normal intestine (left) and the 2019 patient biopsy (right). Reduced staining signal in the patient correlates with altered mitochondrial function. Scale bar: 100 μm.

In 2017, of this patient revealed persistent acute anemia and an intestinal inflammatory process (Figure 1B). Endoscopy of the small intestine revealed several aphthous ulcers and Haematoxylin and eosin (H&E) staining confirmed atrophic and flattened microvilli with reduction of goblet cells, microvilli oedema, cryptitis, and inflammatory infiltrate (Figure 2A). Cytomegalovirus (CMV), which is an opportunistic infection for weakened/immunosuppressed tissue, was also PCR-positive in a CMV culture on the ileal mucosa biopsy, which could explain the observed inflammation (Figure 1B). No other relevant clinical findings were reported after physical examination. However, HIV infection and other causes of immunosuppression studied were negative. The patient was standard treated with a 14-day cycle of intravenous ganciclovir followed by a 14-day cycle of oral ganciclovir. In the 2018 evaluation, biochemical and serological monitoring studies revealed persistent anemia and inflammation without traces of CMV infection (Figure 1B). Evaluation of the biopsy still showed aphthous ulcers in the small intestine and inflammatory infiltrate (Figure 2B). The patient was also treated with intravenous iron, to which he responded positively and restored serum ferritin levels. However, at the most recent monitoring (2019 evaluation), calprotectin and fibrinogen were still altered (Figure 1B), which suggested an intestinal inflammation compatible with ileitis/Crohn's disease. H&E evaluation confirmed the intestinal inflammation (Figures 3A,B). Thus, intestinal inflammation was independent of the CMV infection in the patient. Functional imaging using Tc-99m-tektrotyd-SPECT, which binds to receptors specific for neuroendocrine lesions in the distal ileum (6), showed increased absorption in ileum and peri-ileal lymph nodes that was in agreement with premalignant neuroendocrine lesions in the intestine, similar to those previously observed in the stomach (data not shown).

In order to test a putative relation and possible metaplasia or metastasis from the previously diagnosed gNET, we stained the 2019 biopsy with anti-Chromogranin A antibody, which typically stains hyperplasia of neuroendocrine cells and was found to be increased in gastric tissue of this patient (1). No increased staining was observed in the 2019 biopsy of the intestine, suggesting a primary inflammation in the intestine unrelated to the previously diagnosed gNET.

Immunohistochemistry studies were performed to further characterize the inflammation. The 2019 biopsy was stained with anti-cytokeratin 7 antibody (CK7) biomarker following epithelial inflammatory neoplasms guidelines for expression patterns (7). Multiple anti-CK7 positive foci were observed for this patient (Figure 3C), which indicates inflammatory bowel disease-associated dysplasia, either ulcerative colitis or Crohn's disease (8). On the other hand, the origin of the gNET tumor of this patient was related to an impaired acid-base balance, which was altering mitochondrial function in the stomach (3). To test the involvement of this same mechanism in the new inflammation process, we stained the 2019 biopsy with anti-Pyruvate Carboxylase antibody and compared it to a healthy intestine (Figure 3D). Reduction of staining in the patient's sample corresponds to the lack of mitochondrial function, as we previously observed in the stomach (3). Finally, to evaluate if this observation was specifically found in this patient or whether it was a common trait in all members of the family affected with gNETs, we conducted a non-invasive serological evaluation of patients II.3, II.7, II.8, and II.9 (all gastrectomized) (Table 1). A high sedimentation rate was observed in the gNET patients of the family, which suggested an inflammatory process for these individuals as well. Calprotectin was highly increased in all individuals from the family affected with gNETs, mimicking the described monitoring studies of the proband patient. No traces of CMV infection were observed in these patients. Most relevant biochemical parameters were also tested in healthy siblings of the family without gNETs disease as control. In average, normal values were found for C reactive protein (0.183 C mg/dL), ferritin (47.6 ng/mL), and Sedimentation rate (15.5 mm/h) parameters.

**Table 1:** Pre and post Fe-treatment serology evaluation of the other members of the family affected with gastric neuroendocrine tumors.

**Patient**	**II.3**		**II.7**		**II.8**		**II.9^#x0002A;^**	
**Fe-treatment**	**Pre**	**Post**	**Pre**	**Post**	**Pre**	**Post**	**Pre**	**Post**
Red blood cells [4.50–6 uL]	5.07	5.19	3.76	4.52	3.6	3.6	5.13	NT
Hemoglobin[13–17.5 g/dL]	15.3	14.9	8.9	13.5	11.3	11.3	12.5	13.7
Haematocrit [40–53%]	45.9	46.8	27.8	40.6	35.5	35.5	38.8	42
Ferritin [20–275 ng/mL]	12	54	2	NT	9	70	NT	5
Sedimentation rate [1–20 mm/hour]	13	16	35	NT	5	49	NT	33
C-reactive protein [0–0.50 mg/dL]	0.07	0.03	<0.10	NT	<0.10	0.03	NT	1.44
Fibrinogen [150–500 mg/dL]	319	349	333	NT	284	341	NT	NT
Calprotectin [ <50 ug/g]	NT	140	NT	NT	NT	246	NT	NT

*NT: Not tested*;

* *No Fe treatment*.

*Reference values are showed in brackets*.

In summary, a concurrent inflammatory disease was observed in all patients of the family first diagnosed with an aggressive ATP4A-mutated gNET. Anemia, calprotectin and anti-CK7 staining were in agreement with progression of ileitis-associated neoplasia compatible with inflammatory bowel and Crohn's diseases (8). Autoimmune and inflammatory manifestations occur frequently in patients with primary immunodeficiencies (9). In particular, concomitant gastric and small intestinal inflammatory disorders have been described including celiac disease and more extensive collagenous inflammatory disease (10). In addition, there is a described higher incidence of e.g., inflammatory bowel disease or gastric cancer in patients with Common variable immunodeficiency. However, the associated genetic pathogenesis still remains ambiguous (11). Our results are important since contribute to describe the genetic landscape of these clinical associations. In this work, the inflammation in the intestine was observed as a primary disease and not derived from the gastric neoplasm. Moreover, the same mitochondria-malfunction mechanism responsible for the autoimmune-originated gastric neoplasm in the stomach was also observed in the inflamed intestinal tissue, suggesting that the same impaired acid-base balance mechanism caused by the *ATP4A* mutation might also explain the second inflammatory disease. Study of the other gNETs members of the family also revealed inflammatory disease, which is in agreement with the co-occurrence in all patients of the family carrying the same mutation. In addition, this prospective study is in agreement with the observed autoimmune pathologies concurrent with the gNETs in the second family, where the cumulative effect of two mutations in the *ATP4A* and *PTH1R* genes explained the genetic landscape of the gastric disease and the concomitant autoimmune hypothyroidism and rheumatoid arthritis (4). Thus, our results underscore the important role of *ATP4A* mutations in gNET progression, but also of other concurrent pathologies that must be monitored in these patients to prevent malignant transformation in other tissues.

Finally, CMV infection is usually described in immunodepressed patients and contributes to inflammation. However, no cause of immunosuppression other than the ATP4A mutation was found for this patient. In addition, CMV infection in the proband was not observed in the other members of the family with inflammation, which progressed in the patient even after CMV eradication (Figure 1B). Thus, our data suggest a secondary colonization of the virus not involved in the inflammatory progression but colonizing the injured intestinal tissue. Importantly, secondary opportunistic *H. pylori* infection, which classically contributes to achlorhydria and chronic gastritis, was suggested in patients with gastric achlorhydria (3). Therefore, our results suggest a second pathologic event in the studied family.

The exact molecular mechanism of this histological transformation or the prognostic implications is still unclear. Till then it might be prudent to follow up these patients to assess for the relapse of inflammatory bowel disease as well as for dysplasia surveillance. Perspective follow-up based in mild-moderate ileitis in the context of Crohn's disease guidelines is being carried out for these patients. Evolutionary control is carried out with analytics and periodic termination of calprotectin in feces. Likewise, when the diarrhea symptoms become more intense, we have performed treatment with short courses of oral budesonide as indicated for microscopic colitis (12), with a clear improvement in the symptoms.

## Conclusion

Our results are important since patients with ATP4A mutations or achlorhydria-mediated gNETs have been observed to have secondary pathologies and infections that co-occur with the gastric neoplasm. Long-standing inflammatory bowel disease and CMV infection are premalignant conditions and pose an increased risk of colorectal adenocarcinoma (13) and gastrointestinal neoplasms (14). Therefore, our findings should alert clinicians to monitor patients with gastric neoplasms mediated by mechanisms involving achlorhydria. Further studies in other families affected with gNETs or chronic atrophic gastritis must be performed in order to describe the whole spectrum of concurrent pathologies but these patients must be monitored and careful attention must be paid to other inflammatory diseases that may trigger new severe pathologies.

## Data Availability Statement

The original contributions presented in the study are included in the article/supplementary material, further inquiries can be directed to the corresponding author.

## Ethics Statement

Written informed consent was obtained from the individuals for the publication of any potentially identifiable images or data included in this article.

## Author Contributions

OC and JR obtained the data collection and performed the data analysis and interpretation. OC and JB supervised the study and draft the manuscript. All authors contributed to the article and approved the submitted version.

## Conflict of Interest

The authors declare that the research was conducted in the absence of any commercial or financial relationships that could be construed as a potential conflict of interest.
